# Controversies on Rituximab Therapy in Sjögren Syndrome-Associated Lymphoproliferation

**DOI:** 10.1155/2009/424935

**Published:** 2010-01-28

**Authors:** Luca Quartuccio, Martina Fabris, Sara Salvin, Marta Maset, Ginevra De Marchi, Salvatore De Vita

**Affiliations:** ^1^Rheumatology Clinic, DPMSC, Azienda Ospedaliero Universitaria “S. Maria della Misericordia”, University of Udine, 33100 Udine, Italy; ^2^U.O.C. di Medicina, Presidio Ospedaliero di San Daniele del Friuli, San Daniele del Friuli, 33038 Udine, Italy

## Abstract

Sjögren's syndrome (SS) is a systemic autoimmune disease characterized by chronic inflammation of salivary and lachrymal glands, and frequently accompanied by systemic symptoms. A subgroup of SS patients develops malignant B cell non-Hodgkin's lymphoma (NHL), usually of the mucosa-associated lymphoid tissue (MALT) type and very often located in the major salivary glands. Currently, there is a lack of evidence-based intervention therapy which may influence SS-related chronic inflammation and lymphoproliferation. B cells are involved in the pathogenesis of SS, and B cell downregulation may lead to a decrease of disease activity. Rituximab (RTX), a chimeric monoclonal antibody targeting the CD20 antigen on the B cell surface, has been successfully investigated in other autoimmune diseases, such as rheumatoid arthritis, systemic lupus erythematosus, ANCA-associated vasculitis, and mixed cryoglobulinemic syndrome. Preliminary experiences of RTX therapy in SS patients with or without a lymphoproliferative disorder suggest that SS patients with more residual exocrine gland function might better benefit from RTX. Efficacy of RTX in SS-associated B-cell lymphoma, mainly in low-grade salivary gland lymphomas, remains an open issue.

## 1. Introduction

Sjögren's syndrome (SS) is a systemic autoimmune disease characterized by chronic inflammation of salivary and lachrymal glands, frequently accompanied by systemic symptoms [1]. The presence of various autoantibodies such as the rheumatoid factor (RF) and anti-SSA/SSB antibodies, as well as hypergammaglobulinemia, reflects B cell hyperactivity [1]. SS has the strongest link with non-Hodgkin's lymphoma (NHL) compared with other autoimmune diseases, and similar to mixed cryoglobulinemic syndrome, which may be associated with SS [2–4]. About five percent of patients with SS develop a malignant B cell NHL, usually of the mucosa-associated lymphoid tissue (MALT) type and frequently located in the parotid glands [1, 5]. Currently, there is a lack of evidence-based intervention therapy which may influence SS-related chronic inflammation and lymphoproliferation. In particular, the optimal treatment for NHL complicating SS is not clearly defined. The majority of SS patients with indolent NHL may require only surveillance and no therapy; however, a subgroup of SS patients may suffer from aggressive lymphomas, that is, de novo diffuse large cell B-cell lymphomas, or indolent or low-grade lymphomas progressed into aggressive lymphomas [2]. Although SS has been regarded as T-cell-mediated disease, B cells comprise in general up to 20% of the mononuclear cells in the salivary glands [1, 6]. B-cell activating factor (BAFF) promotes B-cell survival and differentiation, and SS patients frequently have elevated serum levels of BAFF [7]. BAFF overproduction in mouse models results in several autoimmune phenomena, resembling SS and lupus features, as well as in B-cell hyperplasia and lymphoma development [8, 9]. Thus, B cells are involved in the pathogenesis of SS, and B cell downregulation may be a target of treatment. Rituximab (RTX), a chimeric monoclonal antibody direct against the CD20 molecule expressed on the surface of mature B cells, is then a putative therapy for both sicca syndrome and SS-related B-cell lymphoproliferation [10]. Patients with more residual exocrine gland function, for example, those with SS of shorter duration, might better benefit from systemic therapy, as well as SS patients with the cryoglobulinemic syndrome, as reported in recent studies [11, 12]. However, in earliest experience reported between the years 2000 and 2002 by our group, RTX efficacy on nonmalignant lymphoproliferation in SS was inconstant, and a scarce effect on sicca symptoms was observed [13]. Then, a careful use of RTX in selected cases seemed more rationale, in the lack of additional clear-cut evidence of some benefits. By contrast, RTX monotherapy or RTX combined with cytotoxic agents in chemotherapeutic regimens may have a stronger rationale in SS patients with CD20-positive B-cell NHL [3, 11, 14–22].

## 2. Pathologic and Molecular Background, and Involvement of BAFF

Low-grade marginal zone MALT-type lymphoma, usually involving the parotid glands, is an important complication of primary SS [1, 2, 23–25]. A 250-fold increase in risk of parotid gland NHL and a dramatic 1000-fold increase in risk of parotid gland MALT lymphoma were recently observed [2]. However, a positive associations between SS and other subtypes, most notably diffuse large B-cell lymphoma and nodal lymphomas, was reported [2]. Parotid lymphoma may evolve from parotid lymphoepithelial sialadenitis (LESA), which may in turn present with different pathologic and molecular patterns of B cell proliferation, that is, fully benign or with lymphoproliferative lesion by histopathology; and poly-, oligo-, or monoclonal-fluctuating, -persistent or -disseminated by molecular studies [23]. A notable histological feature in SS is LESA characterized by a lymphoid population surrounding and infiltrating salivary ducts, with disorganization and proliferation of the ductal epithelial cells (lymphoepithelial lesions) [26]. These lesions first appear as small clusters and later enlarge to organize lymphoid follicle-like structures with germinal centers. The phenotype of the immunocompetent cells present in lymphoepithelial lesions, mainly composed of primed CD4+ T lymphocytes, suggests functional structures in which activated B cells produce autoantibodies [27–29]. Therefore, LESA is a condition in which MALT is found in the salivary glands, a site not normally containing lymphoid tissue. The MALT acquired due to the autoimmune process may represent the substrate from which the B cell lymphoma develops in SS [1, 23, 30].

The characterization of the earlier events of lymphoproliferation in SS is still ill-defined. Because a monoclonal expansion does not necessarily constitute a malignant lymphoma, monoclonal B cell populations in early LESA represent nonmalignant expansions rather than an early NHL, at least in most cases [23]. The presence of the same B-cell clone in metachronous biopsies of the same affected tissue from the same patient indicates a higher risk of NHL evolution, while different clones in metachronous biopsies are consistent with a nonmalignant B-cell expansion [23, 31]. Thus, the different types of B cell clonal expansion (oligoclonal or monoclonal, smaller or larger, fluctuating or established, localized or disseminated) in SS salivary MALT imply a different risk of lymphoma progression in our experience. Since the transition of LESA into a malignant lymphoma is generally considered to represent a multistep process, molecular studies of clonal B-cell expansion appear of value to better define the risk of lymphoma evolution in SS [23]. It has been speculated that lymphoproliferation is driven by antigenic stimulation, and that during the course of the disease additional oncogenic events occur, such as constitutive activation of signaling pathways after chromosomal translocations or inactivation of tumor suppressor genes.

Cytokine and chemokine microenvironments in target tissues may play a role in the lymphoproliferation in the course of SS. Recently, Barone et al. demonstrated the association between lymphoid chemokines CXCL13 and CCL21 and areas of reactive lymphoid proliferation in the salivary glands with LESA, while CXCL12 was observed predominantly in infiltrated ducts and malignant B cells, suggesting that in salivary gland MALT lymphoma, the lymphoid chemokines CXCL13 and CCL21 are directly implicated in the organization of ectopic lymphoid germinal centers, whereas CXCL12 is associated with the infiltrated epithelium and malignant B cell component and is possibly involved in the regulation of malignant B cell survival [32]. The differential expressions of CXCL13, CCL21, and CXCL12 within SS salivary glands with LESA and MALT-Lymphoma regulate diverse functional microenvironments of lymphoid organization, respectively orchestrating the reactive lymphoid areas that favor the perpetuation of the autoreactive response and the malignant areas where expansion and proliferation of the B cell clones take place [32].

Overproduction of BAFF, implicated in the maturation and survival of B lymphocytes, may play a role in the mechanism of salvage of autoreactive B cell (e.g., RF-positive B-cells) and in the B-cell hyperplasia, as demonstrated in mouse models. Mice carrying a transgene for BAFF become highly susceptible to the autoimmunity-lymphoproliferation complex development [8, 9]. Analysis of BAFF plasma levels in SS has shown that BAFF is higher in patients than in controls and strongly correlates with the titer of autoantibodies such as RF and anti-Ro/SSA [7]. A strong expression of BAFF was also detected in infiltrating lymphocytes, in ductal and acinar epithelial cells in target tissue biopsies from SS patients [20, 33–35]. The prolonged survival of autoreactive B cells may promote production of autoantibodies or the later development of NHL in SS patients. Finally, TNF^−/−^ BAFF transgenic mice also showed a high incidence of B cell lymphomas [36].

RTX exposure increases BAFF overproduction [12, 20]. Recently, we described in detail one of reported SS cases with lymphoma undergoing RTX therapy [20]. In such a case, BAFF overproduction after RTX administration was decreased, but not halted by high-dose steroids (prednisone 1 mg/kg/day for one month). However, BAFF overproduction reappeared at even higher levels after steroid dose reduction. Pre- and post-RTX parotid gland biopsies demonstrated the persistence of BAFF and BAFF-receptor overexpression at the histopathologic and molecular level [20]. Lymphoma in this patient did not respond to RTX therapy, and BAFF was considered as a key resistance factor [20].

In a second case of SS with parotid LESA, who developed rheumatoid arthritis after interferon-alpha therapy for SS, RTX therapy was administered at time of relapse of polysynovitis in a long-term period of follow-up (107 months), with 4 complete cycles of RTX and 6 subsequent single doses of RTX every 6–12 months. Repeated parotid biopsy after 4 full RTX cycles given in 3,5 years revealed a reduction in the parotid lymphoid infiltrate and recovery of acinar and lobular structures at histopathologic level. However, PAN-B and PAN-T antibody stains disclosed a very large amount of B and T cell infiltrates, similar to baseline analysis. Thus, prolonged RTX therapy, that is, at least 4 full cycles, may not affect parotid lymphoproliferation in SS. Pers et al. [37] demonstrated that baseline serum levels of BAFF correlated inversely with the duration of B cell depletion after RTX in SS. B cells were absent in salivary glands analysed 12 months after RTX therapy in sequential lip biopsies, but they were again detected 24 months after RTX. Memory and transitional type 1 (T1) B cells were the first repopulating B cells. Recovery of salivary gland B cells occurred with four B cell subsets: plasmablasts, T1 B cells, mature Bm2 cells, and memory B cells. Increased numbers of Bm2 cells and decreased memory B cells reappeared with time. B cell repopulation in SS glands may be modulated by BAFF and, notably, is followed by reconstitution of the preexisting abnormalities [37].

By integrating the clinical and molecular results herein presented, the concept that BAFF plays a crucial role in B-cell proliferation in the course of SS, at least in a subset of patients, is reinforced, and BAFF tissue overexpression may therefore represent a mechanism of RTX resistance in B-cell lymphoproliferation associated with SS.

## 3. Data from Case Reports and from Open-Label Trials

Since 2002 up to the time of searching (February 2009), 26 patients with SS and lymphoma treated with RTX have been reported in 11 PubMed indexed articles by combining the key words “Sjögren's syndrome”, “rituximab”, and “lymphoma” [3, 11, 14–22]. Sixteen (17/26) were in Ann Arbor Stage I-II, while the remaining were in stage III (1/26) or IV (8/26). Thirteen (14/26) were salivary gland MALT-type lymphomas, mainly parotid gland MALT-type lymphomas (11/26). In 10/26 patients, RTX was administered in combination with other cytotoxic agents (with cyclophosphamide/doxorubicin/vincristine/prednisone (CHOP) regimen in 9 patients, with cyclophosphamide/vincristine/prednisone (CVP) regimen in 1), while in the remaining 16 cases RTX was used in monotherapy (9/26), or with methylprednisolone pulses, low to medium doses of steroids, antimalarials, methotrexate, azathioprine (5/26), or after local radiotherapy (1/26). Globally, response rate to RTX (alone or added to chemotherapy) in SS-related B-cell NHL was 20/26 (77%).

However, while all the ten patients treated with combined therapy with RTX plus cytotoxic agents in chemotherapeutic regimens had a good outcome, response was observed in 10/16 (62,5%) of the 16 cases treated with RTX without cytotoxic therapy. Of the 10 NHLs responding to RTX without chemotherapy, 8 were in I-II Ann Arbor stage (treated with RTX monotherapy) and two were in IV Ann Arbor stage (one also treated with local radiotherapy). Six out of the 10 NHLs responding to RTX without chemotherapy had salivary MALT-type lymphomas, and the other 4 disclosed nonsalivary MALT-type lymphomas. Interestingly, all the nonresponding lymphomas (6/6) involved the salivary glands (Table 1). Thus, salivary gland NHLs, if compared to MALT NHLs involving other organs, seemed more resistant to RTX therapy in the lack of concomitant chemotherapy.

Finally, a higher incidence of human antichimeric antibodies (HACA) and serum sickness reaction was reported after RTX in SS., if compared to other autoimmune diseases [12, 19].

## 4. Personal Experience

Early data on SS patients treated with RTX were presented by our Group in 2002 [13], showing a lack of effect of RTX on nonmalignant parotid monoclonal B-cell lymphoproliferation in LESA. Unexpectedly, lung NHL in one of these two cases showed persistent regression after RTX [12]. At present, we have treated 6 SS patients with RTX in the attempt to improve SS-related manifestations. Indications for RTX were the following: B-cell NHL in 4 patients and nonmalignant parotid lymphoproliferation in 2 patients (LESA; one of them also with cryoglobulinemic syndrome). Other clinical manifestations are described in Table 2, including rheumatoid arthritis in one patient with parotid LESA [38]. RTX was administered at the standard haematological doses, that is, 375 mg/m^2^ weekly for 4 weeks. One patient underwent two subsequent full cycles of RTX, and another patient underwent 4 full cycles and then 6 single (600 mg) RTX infusions at the time of rheumatoid arthritis relapse. Sicca syndrome responded much better in the patient's perspective than in clinical objective evaluation, with about half of the patients reporting an improvement in oral and ocular dryness, while objective tests (i.e., Schirmer test and unstimulated sialometry) remained unchanged or worsened in all the patients. However, constitutional symptoms such as fatigue, low-grade fever, arthralgias, and myalgias, improved in all patients. Parotid swelling decreased or disappeared in 5/6 patients. Cutaneous vasculitis responded in 2/3 patients; sensory peripheral neuropathy improved in 2/3 patients; polysynovitis improved in 3/4 patients. Cryoglobulins and the RF (when present) decreased but did not become negative at month + 6, and anti-SSA/SSB autoantibodies remained positive in all the 6 cases. Lymphoma histotypes and staging were one parotid MALT-type low-grade B-cell NHL (stage IE) [20], one lung MALT-type low-grade B-cell NHL associated with parotid LESA (stage IE) [10, 23], one aggressive diffuse large B-cell NHL associated with salivary MALT-type low-grade B-cell NHL (stage IV), and one parotid gland low-grade MALT-type B-cell NHL with bone marrow NHL infiltration (stage IV). RTX combination therapy with cytotoxic agents was administered in one patient (aggressive diffuse large B-cell lymphoma). One patient underwent monolateral parotidectomy before RTX (parotid gland MALT-type monolateral lymphoma with bone marrow infiltration). Complete response was observed in 3/4 NHL patients, with no relapse in the long-term follow-up (mean survival time 5.5 years). One patient died for right heart failure at month + 39 (secondary to severe mitral and tricuspidal valve insufficiency). Of note, in the patient with lung MALT-type low-grade NHL associated with parotid LESA, biopsy-demonstrated [10, 23], a complete response to RTX monotherapy was observed in lung MALT-type lymphoma, while nonmalignant proliferation (LESA) in the parotid glands persisted [10] with parotid swelling still evident at present.

Treatment with RTX was well tolerated. Serum sickness reactions were not recorded.

Overall, RTX proved effective on SS systemic symptoms, ineffective on sicca syndrome, ineffective on LESA (documented by repeated biopsy) [10, 23], and of value, alone or added to chemotherapy, in SS-related NHL.

## 5. Conclusion

Despite the efficacy of RTX in constitutional and systemic features associated with B cell expansion in SS (i.e. cryoglobulinemic vasculitis) [12], the usefulness of RTX in B-cell lymphoproliferation in SS is controversial. Aggressive lymphomas require standard chemotherapeutic regimens, which were recently integrated with RTX [15]. However, the majority of SS patients with lymphoma show indolent, nonaggressive lymphomas, mainly salivary (parotid) MALT-type lymphomas in stage I-II, with a performance status 0-1 [39]. An aggressive approach would be more dangerous rather than curative in this subset. Parotid MALT-type NHL associated with SS appears to be to be more resistant to RTX as compared with the other types of lymphoma in SS, and RTX may be as well ineffective in SS-related LESA. RTX may fail to deplete B-cells in the salivary tissue due to key pathogenetic factors in the local microenvironment, as recently well described in a murine model [40]. A still unknown local trigger is also suspected. Local factors such as BAFF and chemokines (CXCL13, CCL21, CXCL12) appear to be implicated, and BAFF inhibition plus anti-CD20 therapy allowed reaching much higher degrees of B-cell depletion in mouse models [40].

Thus, by integrating clinical and molecular data, RTX monotherapy may not represent an ideal treatment for SS-associated lymphoproliferation, and B-cell recovery after RTX, when present, showed the restoration of previous B-cell abnormalities [37]. Combining RTX with cytotoxic agents in aggressive lymphomas, or with the recently available anti-BAFF agents in indolent lymphomas and prelimphomatous conditions (e.g., persistent parotid swelling with ongoing monoclonal B-cell expansion, and/or type II mixed cryglobulinemia) could prove more effective, and dedicated studies are needed. Persistence of antigen-driven stimulation of the immune system in the target tissue may finally require an etiologic treatment, as in gastric low grade NHL [41].

## Figures and Tables

**Table 1:** Patients with SS and lymphoma treated with rituximab.

Author, years	N. of pts	Type of lymphoma/bone marrow involvement	Ann Arbor Stage	Response on lymphoma	Other concomitant treatments for lymphoma
Shih, 2002	1	Parotid gland MALT-type/no	IE	Yes	No

Somer, 2003	1	Parotid gland MALT-type/no	IE	Yes	No

Voulgarelis, 2004*	4	(1) Salivary gland MALT-type/yes*	IV	Yes	(1) CHOP
		(2) Nodal marginal zone/no*	IIE	Yes	(2) CHOP
		(3) Pulmonary MALT-type/yes*	IV	Yes	(3) CHOP
		(4) Salivary gland MALT-type/yes*	IV	Yes	(4) CHOP

Harner, 2004	1	Nodal marginal zone/Pulmonary MALT-type/no	IIE	Yes	No

Ramos-Casals, 2004	2	(1) Ovarian MALT-type/yes	IV	Yes	(1) CHOP
		(2) Ocular MALT-type/yes	IV	Yes	(2) Local radiotherapy

Pijpe, 2005	1	Parotid gland MALT-type/no	IE	Yes	No

Gottenberg, 2005^#^	2	(1) Digestive tract MALT-type/no^#^	IE	Yes	(1) MP 500 mg × 4, HQ
		(2) Salivary gland MALT-type/no^#^	IE	No	(2) No

Pijpe, 2005	7	(1) Parotid gland MALT-type/no	IE	No	(1) No
		(2) Parotid gland MALT-type/no	IE	No	(2) PDN 15 mg/day
		(3) Parotid gland MALT-type/no	IE	Yes	(3) No
		(4) Parotid gland MALT-type/no	IE	Yes	(4) No
		(5) Parotid gland MALT-type/no	IE	No	(5) PDN 7.5 mg/day, MTX
		(6) Parotid gland MALT-type/no	IE	Yes	(6) PDN 5 mg/day, AZA
		(7) Parotid gland MALT-type/no	IE	No	(7) No

Voulgarelis, 2006*	6	(1) Nodal marginal zone/no	II	Yes	(1) CHOP
		(2) DLBCL/no	II	Yes	(2) CHOP
		(3) Salivary gland MALT-type/yes*	IV	Yes	(3) CHOP
		(4) Nodal marginal zone/no*	IIE	Yes	(4) CHOP
		(5) Pulmonary MALT-type/yes*	IV	Yes	(5) CHOP
		(6) Salivary gland MALT-type/yes*	IV	Yes	(6) CHOP

Seror, 2007^#^	5	(1) Salivary gland MALT-type/no^#^	IE	No	(1) No
		(2) Nodal marginal zone/yes	IV	Yes	(2) No
		(3) Gastric/pulmonary MALT-type/no	IV	Yes	(3) Mini-CHOP
		(4) Gastric MALT-type/no^#^	IE	Yes	(4) HQ
		(5) DLBCL/no	III	Yes	(5) CHOP

Quartuccio, 2008°	1	Parotid gland MALT-type/no°	IE	No	High-dose steroids

Carbone J, 2008	1	Low-grade marginal zone/yes	IV	Yes	CVP

Present report°	4	Parotid gland MALT-type/no°	IE	No	High-dose steroids
		Lung MALT-type /no	IE	Yes	No
		DLBCL + salivary MALT-type/no	IV	Yes	CNOP
		Parotid gland MALT-type/yes	IV	Yes	Parotidectomy

Legend: pts, patients; MALT, mucosa-associated lymphoid tissue; CHOP, cyclophosphamide, doxorubicin, vincristine, prednisone; AZA, azathioprine; PDN, prednisone; MTX, methotrexate; MP, methylprednisolone; DLBCL, diffuse large B-cell lymphoma; HQ, hydroxychloroquine.

*There is some patients' overlapping between the cases reported in Voulgarelis, 2004 and Voulgarelis, 2006.

^#^There is some patients' overlapping between the cases reported in Gottenberg, 2005 and Seror, 2007.

°There is some patients' overlapping between the cases reported in Quartuccio, 2008 and present report.

**Table 2:** Clinical and demographic features of our six SS patients treated with rituximab.

Pt.	Age*/ sex	Age at SS diagnosis	Age at NHL diagnosis	Anti-SSA/SSB positivity	SS criteria	HCV	RF positivity	Cryo-Ig(pos/type)	CM	Low C4	Persistent parotid swelling	B symptoms	Vasculitis	BM VDJ restriction	Extraglandular manifestations
1	50/F	31	50	SSA/SSB	I, II, III, IV, V, VI	No	No	No	No	No	yes, bilateral	No	No	polyclonal	Arthralgias, fatigue, fibromyalgia
2	59/F	55	59	SSA/SSB	I, II, III, IV, V, VI	No	yes	Type II	IgM-k	yes	yes, bilateral	No	yes	oligoclonal	Neuropathy, ILD, arhralgias, fatigue
3	47/F	41	47	SSA/SSB	I, II, III, V, VI	No	yes	Type II	IgM-k	yes	yes, bilateral	No	yes	polyclonal	Neuropathy, arthralgias, fatigue
4	42/F	41	42	SSA/SSB	I, II, III, V, VI	No	yes	Type II	IgM-k	yes	yes, monolateral	No	No	monoclonal	Thyroiditis, arthralgias, fatigue
5	54/F	46	n.a.	SSA/SSB	I, II, III, IV, V, VI	No	yes	No	No	No	yes, bilateral	No	No	polyclonal	Erosive arthritis, fatigue
6	59/F	57	n.a.	SSA	I, II, III, V, VI	No	yes	Type II	IgM-k	yes	yes, bilateral	No	yes	polyclonal	Raynaud, neuropathy, arthritis, fatigue

Legend: SS: Sjögren's syndrome; HCV: hepatitis C virus; NHL: non-Hodgkin lymphoma; anti-SSA/SSB: anti-ENA antibodies with SSA/SSB specificity; RF: rheumatoid factor; cryo-Ig: serum crioglobulins; MC: monoclonal component; ILD: interstitial lung disease; BM VDJ: bone marrow variable, diversity and joining region rearrangement; n.a.: not applicable.

*age at RTX therapy.
